# Supplementary material for: Are carbon emissions trading and green financial instruments synergistic? -Comprehensive quantitative research based on content analysis
Source: PLoS One. 2024 Mar 7;19(3):e0298601. doi: 10.1371/journal.pone.0298601 (PMC10919601; doi:10.1371/journal.pone.0298601)
Supplement: S1 Data — (ZIP) [file pone.0298601.s001.zip › Data/地级市Z_F工作报告.zip!/░▓╗╒/╗╞╔╜╩╨/└┤╫╘╣1⁄2╓┌║┼í╛┬φ┐╦╩2╛▌═°í┐_2019.docx]

各位代表：

现在，我代表市人民政府，向大会报告政府工作，请予审议，并请市政协委员和其他列席人员提出意见。

**一、2018年工作回顾**

过去的一年，面对错综复杂的宏观环境，全市上下在省委、省政府和市委的坚强领导下，坚持以习近平新时代中国特色社会主义思想为指导，贯彻落实党的十九大和十九届二中、三中全会精神，坚持稳中求进工作总基调，坚持以新发展理念为引领，按照高质量发展要求，深入推进供给侧结构性改革，大力实施五大发展行动计划，着力打好三大攻坚战，全面做好“六稳”工作，保持了经济持续健康发展和社会大局稳定，较好完成了市七届人大一次会议确定的目标任务，实现了新一届政府工作的良好开局。

——经济运行总体平稳、稳中有进。初步核算，全市生产总值增长7.5%左右，财政收入增长7.5%，固定资产投资增长15%，社会消费品零售总额增长11%，外贸进出口总额增长24.8%。

——基础设施建设实现重大突破。国家重大水利工程月潭水库即将建成蓄水。杭黄高铁建成通车，昌景黄高铁安徽段和池黄、宣绩（黄）高铁关键性工程及黄千高速、德上高速开工建设，我市正加快打造成为全国交通网络的重要节点、皖浙赣区域综合交通枢纽城市和皖南现代化区域性中心城市。

——改革开放迈出重大步伐。全面落实省委省政府积极推动长三角高质量一体化发展决策部署，成功加入杭州都市圈，与杭州市签署“1+9”战略合作协议，黄山与杭州等地建构了从“共饮一江水”迈向“共享一个圈”的经济新形态。大力推进“互联网+政务服务”，18种常见不动产登记业务最快1小时办结，跑出了“安徽最快速度”。

——“新安江模式”在全省乃至更大范围推广。省委书记李锦斌历时三天、行程千里，考察调研新安江全流域，省长李国英多次深入实地考察指导，省委省政府专门召开系列会议、出台系列文件，全面总结推广新安江流域生态补偿机制试点经验。试点工作写入党中央国务院《关于建立更加有效的区域协调发展新机制的意见》，入选全国“改革开放40年改革创新40案例”、安徽省改革开放40周年重点领域改革八大品牌，“新安江模式”成为习近平生态文明思想的积极探索和生动实践。

——全域旅游发展势头强劲。积极推进“旅游+”战略，强力推进旅游“品质革命”，深入实施全域旅游发展规划，全年乡村旅游接待4725万人次，占全市游客接待量的三分之二以上，山上山下联动、观光休闲并重的全域旅游格局加速形成，打响了“中国旅游，从黄山再出发”品牌。

——人民生活持续改善。城镇和农村常住居民人均可支配收入分别增长8.7%、9%；居民人均储蓄存款余额5.3万元，位居全省第一。脱贫攻坚再战再捷，11739名贫困人口稳定脱贫，贫困发生率降至0.17%。人民群众获得感、幸福感、安全感进一步增强。

一年来，主要做了以下工作：

（一）精准施策出实招，经济保持平稳增长。出台促进经济高质量发展、加快建设创新型黄山、降低企业成本、促进民营经济高质量发展“1+10+N”等系列政策措施，严格落实市级领导联系重点企业、重点项目等制度，常态化开展“四送一服”双千工程，全力优化经济发展环境。全年安排扶持实体经济发展财政资金5.44亿元，减免企业税费16.86亿元，落实银企对接资金179.85亿元，解决企业过桥贷款43.08亿元，新型政银担业务贷款余额44亿元。严格落实“四督四保”制度和“五项机制”，组织开展“重大项目攻坚月”活动，全年完成重点项目投资345.7亿元，235个项目纳入省稳投资项目库、总投资额1231亿元，争取国家、省项目资金7.89亿元。

（二）坚持不懈调结构，产业升级步伐加快。制定出台乡村振兴战略实施意见，全年农业增加值增长3%，规上农产品加工企业产值增长4%。茶叶一产产值34.28亿元，小罐茶业有限公司跻身中国茶叶品牌传播力和中国茶企双“十强”，“祁门红茶”公用品牌连续9年荣膺中国“品牌价值十强”。山泉流水养鱼综合产值达4亿元。新增家庭农场171家、省级产业化示范联合体11家。成功举办第三届中国休闲农业（黄山）峰会。农产品电子商务交易额突破14亿元。组织实施新型工业化“5542”行动计划，规上工业企业增加值增长10%，市级队工业企业产值增长12%。

战略性新兴产业产值、高新技术产业增加值均增长17%。新增国家高新技术企业22家、院士工作站1家、省高层次人才团队2个。全市开发区规上工业总产值增长20%，税收增长17.8%，7个开发区全部去筹转正，市经济开发区成功创建省高新技术产业开发区，歙县经济开发区进入国家级开发区培育名录。黄山现代服务业产业园核心区累计完成投资14亿元，文创小镇开园运营；全市服务业增加值增长7%。扎实推进皖南国际文化旅游示范区建设，全面加快黄山东部开发、月潭湖综合开发，规划建设新安江百里大画廊和“皖浙1号线”等十条风景道，全年游客接待量6487万人次、旅游总收入572亿元，分别增长12.3%和13.2%，其中入境游客263万人次、增长10.6%，过夜登记游客近千万。

（三）深化改革扩开放，发展活力日益增强。隆重举行庆祝改革开放40周年系列活动。着力创优“四最”营商环境，深入实施“放管服”改革，扎实开展“减证便民”专项行动，全面取消自行设立证明事项。全面推行“一窗”分类受理，企业投资项目实现开工前审批“最多100个工作日”，一网通办事项占比96.3%，市本级政务服务事项“最多跑一次”实现率99.26%。深化商事制度改革，企业开办时间压缩到3个工作日内，新增市场主体1.8万户、增长17.2%。深化综合医改，公立医院薪酬制度、医疗服务综合监管等改革取得突破。持续深化城管执法体制改革，旅游管理领域相对集中行政处罚权试点全面推开。组建黄山铁投集团、交投集团，黄山旅游集团蝉联中国旅游集团20强。深化农村综合改革，完成集体资产清产核资工作和217个村“三变”改革、280个村股份合作制改革，黄山区入选国家级农村综合性改革试点，黟县入选全国第三批农村集体产权制度改革试点县。全年新增“新三板”挂牌企业3家、省股权托管交易中心挂牌企业36家，总规模40亿元的安徽省专精特新基金落户我市。举办黄山旅游节、联合国教科文组织黄山对话会等重大活动，黄山风景区再获桂冠，成功加入世界生物圈保护区网络。深化与“一带一路”沿线国家和地区合作，加强与瑞士因特拉肯市、瑞典瓦拉市等友好城市交往，与肯尼亚梅鲁郡签订友好交流备忘录，“梦幻黄山·礼仪徽州”形象宣传片登陆纽约时报广场。黄山口岸出入境8.6万人次，进境水果指定口岸建设通过省级预验收，休宁县茶叶出口基地被评为国家外贸转型升级示范基地。加强与长三角、京津冀对接合作，黄山与武夷山旅游营销一体化加快推进。组团参加首届中国国际进口博览会、世界制造业大会，与华为、科大讯飞等数字经济领军企业签订战略合作协议，众泰汽车智能网联生产基地开工建设，中科创新广场、上海城投高铁CBD、歙县军民融合产业园等项目成功签约，全年新签内资项目532个，其中亿元以上项目140个；到位资金242亿元、增长15%。

（四）建管并重提品质，城乡面貌明显提升。扎实推进全国“城市双修”试点、“两治三改”三年行动，改造提升中心城区道路30条，开工建设黎阳桥、八一大桥，加快推进地下管廊、公共停车场、排水防涝设施、污水厂提标建设和雨污分流改造，实施屯溪老街滨江西路综合提升、花溪饭店地块改造和“徽州坊”文化旅游综合体建设，市生活垃圾综合处理厂建成运营，市资源循环利用基地入选国家资源循环利用基地。歙黟一级公路基本建成，徽屯公交实现一体化互联互通。全面启动新一轮全国文明城市创建，扎实推进“六城联创”。实施休宁县城市基础设施及水环境综合整治PPP项目，歙县中心城区建设取得积极进展，黟县、祁门县老城区改造提质提速，全市城镇固定资产投资增长4.6%。统筹推进农村“三大革命”和农村环境“六治理”，开工建设农村污水治理PPP项目，农村垃圾治理PPP项目全域运营，美丽乡村建设实现皖南片区“四连冠”，黄山区、徽州区、休宁县获评全省美丽乡村建设先进县。

（五）保护传承增特色，生态文化优势彰显。扎实做好中央环保督察反馈意见和“回头看”交办信访件整改落实，着力打好蓝天、碧水、净土保卫战，实施烟花爆竹禁限放管理规定，空气质量在国家重点监测的169个城市中稳居第二。制定实施《新安江生态经济示范区规划》，第三轮流域生态补偿协议签订实施，第十六届中国水论坛暨第二届新安江绿色发展论坛成功举办。严格落实河湖长制，全面推广“生态美超市”，强力推进新安江入河排污口整治；基本建成农药集中配送体系，高效低残和生物农药使用率提高到80%。全面落实林长制，深入开展林业增绿增效行动，认真实施《黄山市松材线虫病防治条例》，坚决守护黄山松安全。扎实开展全国首批历史建筑保护利用试点，新增中国传统村落179个。实施徽剧振兴工程，举办徽班进京故宫展演、故宫博物院藏文房用具暨徽州贡品展，故宫文化创意馆徽派传统工艺馆开馆，徽州大剧院改造完成，市图书馆跻身国家一级馆，新增国家级非遗传承人13人。8家企业入选全省民营文化企业百强，全市重点文化产业项目完成投资90亿元，文化产业增加值增长10%。

（六）全力以赴惠民生，社会保持和谐稳定。精心组织实施33项民生工程，完成投资45.72亿元。城镇新增就业2.62万人，城镇登记失业率控制在2.8%以内。五项社会保险参保人数96.6万人次，城乡居民基本养老保险参保78.87万人。企业退休人员退休费人均月增加126.6元，城市特困人员救助供养和农村特困人员集中供养、分散供养补助标准分别提高6%、8%、10%，累计为16648名困难残疾人发放生活补贴1244.1万元。新农合参合率100%。我市与杭州都市圈城市实现就医结算“一卡通”。发放城乡医疗救助资金5317.8万元。住房公积金惠及职工10.1万人。完成棚户区改造1372套，改造老旧小区53个。治理地质灾害隐患点43处，509户、1687人消除地质灾害威胁。实施打赢脱贫攻坚战三年行动，纵深推进“重补促”专项行动，深入开展“五级书记”遍访贫困对象推进乡村振兴和“万名党员干部帮万户”“百企帮百村”行动，强力推进脱贫攻坚“十大工程”，累计投入资金10.2亿元，提前两年完成易地扶贫搬迁任务，一批贫困村正加快嬗变为美丽乡村。面向全国部分高校引进人才50名，新增省级博士后工作站2家、省级以上技能大师工作室3个，我市选手在第45届世界技能大赛全国选拔赛上荣获3项冠军。全市新改扩建幼儿园8所，屯溪区百鸟亭小学建成招生，全市义务教育阶段学校大班额全面消除，义务教育巩固率达100%，普通高中和职业院校办学条件进一步改善。市委党校新校区建成启用。黄山学院高质量通过教育部本科教学工作审核评估。扎实推进健康黄山建设，加强重大疾病防控和基本公共卫生服务，市人民医院与中日友好医院缔结友好关系，市域内就诊率达93%。落实全面两孩政策，人口自然增长率3.4‰。我市运动健儿在第十四届省运会、第七届省残运会上取得好成绩，龙舟运动员王丽在雅加达亚运会上获一金一银。完成新一届村委会、社区居委会换届选举，新增美丽社区12个。全面落实安全生产责任制，持续开展“铸安”行动，全市安全生产形势总体平稳。强化食品药品安全监管，妥善处置长春长生不合格疫苗问题。严密防控非洲猪瘟疫情。深入开展“七五”普法，扎实开展信访突出问题“深重促”专项行动，市联合接访中心建成启用。深入开展扫黑除恶专项斗争，群众安全感指数位居全省第二。聚焦新时代强军目标，扎实推进练兵备战和民兵调整改革，圆满完成年度征兵任务，国防动员和后备力量建设不断加强。军民融合深度发展，退役军人事务管理、国防教育和人民防空工作取得明显实效，我市荣膺全省双拥模范城“七连冠”。哲学社会科学、新闻出版、广播电视、文学艺术、档案方志、统计等工作继续加强，民族宗教、外事侨务、对台、妇女儿童、老龄、残疾人、红十字事业实现新发展，气象、地震、防灾减灾等工作取得新进步。

（七）提质提效优服务，自身建设不断加强。坚持党对一切工作的领导，始终把政府系统政治建设摆在首位，树牢“四个意识”，坚定“四个自信”，坚决维护习近平总书记党中央的核心、全党的核心地位，坚决维护党中央权威和集中统一领导。全面加强意识形态工作，深入推进“两学一做”学习教育常态化制度化，扎实开展“讲严立”专题警示教育，持续深化“三查三问”。全面落实法治政府建设实施纲要，严格依照法定程序和权限履行职能，完成新一届市政府法律顾问聘任。制定实施全国地级市首部专项规章《黄山市河湖长制规定》，颁布施行《黄山市促进美丽乡村建设办法》。自觉接受市人大及其常委会的法律监督、工作监督和市政协的民主监督，162件人大代表建议、299件政协提案全部办结。深入推进机关效能建设，实施政府服务提质提效工程，全面开展“大调研大督查大落实”活动，政府热线服务平台办理有效群众诉求7.9万余件，办结率100%。严格执行中央八项规定实施细则精神和省委、市委有关要求，全市“三公”经费支出下降3.6%。认真落实党风廉政建设主体责任，深入推进政府系统党风廉政建设和反腐败斗争，加强重点领域和关键环节监管，切实抓好政务公开和政府信息公开，强化审计监督和财政资金监管，确保权力始终在阳光下运行。

各位代表！

过去的一年，我们过得很充实、走得很坚定！在新时代高质量发展的征程上，我们砥砺前行、迎难而上，努力奔跑、奋斗追梦，取得了来之不易、令人振奋的成绩。这是省委省政府和市委坚强领导、科学决策的结果，是市人大及其常委会和市政协有效监督、大力支持的结果，是全市人民撸起袖子加油干、一起拼搏一起奋斗的结果！在此，我代表市人民政府，向全市人民，向各民主党派、工商联和无党派人士、各人民团体、社会各界人士，向驻黄解放军指战员、武警官兵、政法公安干警和消防救援队伍指战员，向关心支持黄山改革发展的中央、省驻黄单位和海内外友好人士，向在我市创业的投资者、建设者，致以崇高的敬意，表示衷心的感谢！

在肯定成绩的同时，我们也清醒地看到，政府工作离广大人民群众的期待仍有差距，经济社会发展中不平衡不充分问题还有不少：经济总量偏小，产业层次不高，生态文化优势尚未充分发挥；科技创新氛围不浓、动能不足，土地、资金、人才等要素制约还未有效破解，部分企业生产经营比较困难；财政收支压力大，基本公共服务供给不足，就业、教育、医疗、养老、住房等民生领域还存在不少短板，生态保护、防灾减灾、安全生产和社会治理任务依然艰巨；运用新发展理念创新性开展工作的能力和水平还不高，庸懒散拖现象还不同程度存在，一些领域不正之风和腐败现象还时有发生。对此，我们一定高度重视，采取切实措施，认真加以解决，不辜负各位代表和全市人民的期望和重托。

**二、2019年主要工作**

2019年是新中国成立70周年，是全面建成小康社会关键之年。做好今年政府工作，意义重大、责任重大。一方面要看到，外部环境复杂严峻，经济面临下行压力，不稳定不确定因素增多。另一方面更要看到，国家继续实施积极的财政政策和稳健的货币政策，我国新一轮改革开放的新战略、新举措正在释放巨大红利，中央和省围绕“六稳”，在基础设施领域补短板、促进消费扩容升级、稳定外资外贸等方面，陆续出台一系列重大政策措施，为我们发挥旅游、文化、生态“三位一体”优势，推进经济高质量发展提供了有力支撑；中央高度重视民营经济和小微企业发展，实施更大规模的减税降费，持续加大金融等支持力度，为我们发展实体经济、构建现代化产业体系创造了更加有利的政策环境；黄山现代综合交通体系日益完善，特别是我市正式加入杭州都市圈，为我们承接产业转移、参与区域分工合作提供了新的机遇。只要我们坚定信心不动摇，咬定目标不放松，以新理念新坐标推动各项工作，就一定能够战胜各种困难和挑战，迎来更加光明的发展前景。

今年政府工作的总体要求是：坚持以习近平新时代中国特色社会主义思想为指导，深入贯彻落实党的十九大和十九届二中、三中全会精神，按照省委经济工作会议和市委六届七次全会部署，坚持稳中求进工作总基调，坚持新发展理念，坚持推动高质量发展，坚持以供给侧结构性改革为主线，坚持深化市场化改革、扩大高水平开放，高质量推进五大发展行动，加快推进新型工业化和“旅游+”，继续打好三大攻坚战，统筹推进稳增长、促改革、调结构、惠民生、防风险、保稳定工作，进一步稳就业、稳金融、稳外贸、稳外资、稳投资、稳预期，增强人民群众获得感、幸福感、安全感，保持经济持续健康发展和社会大局稳定，为全面建成小康社会收官打下决定性基础，加快建设更美丽更富裕更文明的现代化新黄山，以优异成绩庆祝中华人民共和国成立70周年。

主要预期目标是：全市生产总值增长7.5%左右，财政收入增长7%，固定资产投资增长9%，社会消费品零售总额增长10.5%，城镇常住居民人均可支配收入增长8.5%，农村常住居民人均可支配收入增长9%，居民消费价格涨幅控制在3%左右，万元生产总值能耗、主要污染物排放总量控制在省政府下达目标以内。

各位代表，大道至简，实干为要。今年市政府重点做好八个方面工作：

（一）全方位深化改革扩大开放。

全面贯彻落实习近平总书记在庆祝改革开放40周年大会上的重要讲话精神，在更高起点、更高层次、更高目标上推进改革开放。

大力度推进重点改革。全面完成政府机构改革任务。深化“放管服”改革和商事制度改革，全面推行“证照分离”“照后减证”，加大“双随机”跨部门联合抽查力度。深化“互联网+政务服务”，深入推进“一网一门一次”改革，实施市政务服务中心扩容改造工程，加强政务服务标准化建设，实现政务服务事项“应进必进、一门通达”。稳妥推进相关区域和领域财政事权和支出责任划分改革，提高财政资金使用绩效。全面完成市直单位经营性国有资产集中统一监管改革，完善市属国有企业现代企业制度，推动市属国有企业提高发展质量和效益，不断提升对全市经济发展的支撑力和贡献度。健全完善市县公立医院现代医院管理制度，推动公立医院治理体系和管理能力现代化，让人民群众看病更放心、更贴心。

毫不动摇推动民营经济发展。深入贯彻落实习近平总书记在民营企业座谈会上的重要讲话精神，全面落实中央及省、市促进民营经济发展意见措施，组织实施“十大工程”，着力创优“四最”营商环境。严格落实国家和省减税降费政策，继续执行阶段性降低社保费率政策，切实降低企业制度性交易、用工、用地、用能、物流、融资等成本。市财政设立1亿元民营企业过桥纾困资（基）金，统筹整合设立规模2亿元民营企业发展专项资金、3000万元不良贷款担保偿债准备金。鼓励民间资本参与基础设施建设和公用事业发展。严格落实各级领导联系民营企业制度，加快建立营商环境评价机制和企业参与涉企政策制定机制，积极构建亲清新型政商关系。加强民营企业家队伍建设，大力弘扬企业家精神，依法保护企业自主经营权和企业家人身财产安全，让广大民营企业家吃下定心丸、安心谋发展！

全方位融入杭州都市圈。落实全面融入杭州都市圈实施意见和黄杭两市“1+9”战略合作协议，建立健全常态化长效化工作机制，主动对接都市圈新一轮总体规划和系列专项规划，加快建立与都市圈城市等高接轨的政策体系，全力推进黄杭同城化建设步伐。加快交界区域干线公路建设，积极推动新安江干线航道综合开发利用等项目前期工作，协同建设一体化智慧交通管理体系，共同打造以高铁、高速、航运为重点的立体交通走廊。加强产业协作，鼓励我市企业与都市圈城市企业建立产业协同联盟，深化黄山经济开发区与杭州经济技术开发区合作，探索借鉴“反向飞地”等新模式，积极推进特色园区、楼宇经济互促共建。加强与都市圈城市“大院大所”政产学研合作，积极吸引高层次人才、高校毕业生来黄山创新创业。推动成立都市圈旅游营销联盟，联合推广黄杭世界遗产游、名城名山名江名湖名村游等精品线路，共同打响“黄杭黄金旅游带”等区域品牌。协同推进新安江-千岛湖国家生态补偿机制示范区建设，全面深化流域水资源和生态环境保护合作，共建都市圈西部绿色廊道。积极推进都市圈市民卡“一卡通”，加强教育、医疗、文化、体育等各方面合作，加快建成更高水平的基本公共服务体系。

加快打造内陆开放新高地。积极对接京津冀协同发展、长江经济带发展、粤港澳大湾区建设等国家战略，全面参与长三角一体化区域分工合作，积极争取加入长三角城市经济协调会。进一步密切与国际组织的联系，推进与国际友城、“一带一路”沿线国家和地区在旅游、文化、经贸等领域合作。组团参加中国国际进口博览会、世界制造业大会等重大经贸活动。支持企业开拓新的出口市场，引导企业扩大先进设备、重要原材料进口，进境水果指定口岸通过国家验收并投入运营，外贸进出口总额增幅与全省保持同步。

（二）深入实施供给侧结构性改革。

实施供给侧结构性改革是提高经济发展质量和效益的治本之策，要在落实“巩固、增强、提升、畅通”八字方针上采取有力有效措施，注重以“破、立、降”并举巩固“三去一降一补”成果，注重以市场化改革和法治化营商环境增强微观主体活力，注重利用技术创新和规模效应提升产业链水平，注重以加快建设现代市场体系畅通经济循环，实现我市经济高质量发展。

大力推进五大发展行动计划。坚持把五大发展行动计划作为供给侧结构性改革的主抓手，持续推进25项重点任务、55个重点工程，着力增强发展的动力和活力。全力抓好366个市级重点项目建设，力争新开工100个以上、竣工70个以上，完成重点项目投资330亿元以上。基础设施方面，积极实施《黄山铁路地区总图规划》，全面提速昌景黄高铁、池黄高铁、黄千高速、德上高速、芜黄高速、新安江街口大桥等项目建设，加快推动屯溪国际机场迁建、杭临（绩）黄高铁、黄衢高铁、北外环高速、市域旅游铁路等前期工作，建成宁国-黄山天然气支线工程、新城区综合管廊、歙县金山至县城公路。产业发展方面，加快推进众泰汽车智能网联生产基地、小罐茶运营总部基地、新安江百里大画廊和“皖浙1号线”等十条风景道及配套服务设施建设，开工黄山电器新型功率器件迁建入园等项目，确保月潭湖景区年内对外运营。积极谋划亿元以上项目100个，充实完善五大发展行动计划项目库，加快形成“开工一批、竣工一批、谋划一批、储备一批”的梯次推进格局。

全力抓好招商引资。坚持引资引智引技并举，按照“产业+区域”的要求，全面深化与北京市国资委、上海市国资委和央企、知名民企、外企的合作，推进上海城投高铁CBD等一批招商项目开工建设，力促复星文旅、康美药业等项目签约落地，力争在引进数字经济、智能制造、文化创意、度假养生、先进医疗、优质教育等项目上取得重大突破。继续推进小分队招商，加强与商会协会、科研院所合作，探索人才+资本、技术+资本、收购兼并式股权招商和产业引导基金招商，大力开展存量资产、低效闲置土地招商。全面推行投资项目全程代办制、容缺办理制，提高招商项目履约率、资金到位率，全年新签亿元以上项目超120个，实际到位资金增长10%，外商直接投资增长5%。

强化金融支撑保障。深化政银企合作，引导银行业金融机构加大对民营企业和小微企业的信贷支持，力争金融机构贷款余额超900亿元，政策性融资担保贷款余额超45亿元。积极争取扩大地方债、企业债发行规模，推进黄山旅游集团境外债发行，加大专精特新基金投放力度，加快设立新安江国元种子基金、文化旅游及民营经济发展基金，鼓励有条件的企业争取上交所科创板上市，支持黄山旅游、永新股份等上市企业加大股权融资和再融资，力争新增主板上市辅导企业2家、“新三板”挂牌企业2家以上、省股权托管交易中心挂牌企业20家，直接融资突破30亿元。大力发展普惠金融，打造农村资金互助升级版。

（三）坚决打好三大攻坚战。

针对突出问题，打好重点战役，努力为高质量全面建成小康社会打下坚实基础。

打好防范化解重大风险攻坚战。扎实推进防范化解金融风险三年行动计划，持续开展金融机构不良资产压降行动，妥善处置“僵尸企业”直接债务，严厉打击恶意逃废债行为。组织开展打击非法金融机构和非法金融业务专项行动，深入排查涉嫌非法集资、类金融机构资金链断裂风险，扎实推进互联网金融风险专项整治，严厉查处金融违法犯罪行为，坚决守住不发生系统性金融风险底线。加强政府债务管理，规范举债融资行为，严控债务增量，稳妥化解存量，妥善处理政府隐性债务风险，做到坚定、可控、有序、适度。

打好精准脱贫攻坚战。坚持把提高脱贫质量放在首位，扎实推进打赢脱贫攻坚战三年行动。认真抓好中央脱贫攻坚专项巡视及省委巡视反馈的脱贫攻坚领域问题整改，持续开展扶贫领域作风问题专项治理。深入实施脱贫攻坚“十大工程”，大力推进产业扶贫，全面推广“四带一自”“三有一网”模式，因地制宜发展适应性产业。大力推广“一自三合”模式，多渠道发展壮大村集体经济。坚持扶贫与扶志、扶智相结合，深化“三业一岗”就业扶贫，不断扩大就业脱贫覆盖面。全面完成贫困户危房改造任务，实施路网完善、电网扩容、农村饮水安全巩固提升工程，认真落实“351”“180”等政策，切实提高“三保障”水平。加大收入水平略高于建档立卡贫困户群体的帮扶力度。切实加强革命老区脱贫攻坚。继续开展“万名党员干部帮万户”“百企帮百村”行动，巩固脱贫成效，防范返贫致贫，再实现脱贫1800人以上，力争脱贫攻坚工作稳居全省第一方阵。

打好污染防治攻坚战。认真抓好中央生态环保督察“回头看”交办信访件整改工作，积极创建国家生态文明建设示范市。深入开展新安江流域生态补偿机制第三轮试点，用好新安江绿色发展基金，加快建设新安江生态经济示范区，着力打造“长效版”“拓展版”“推广版”。全面落实河湖长制，扎实推进太平湖和阊江流域水环境综合治理，继续实施湿地保护、中小河流治理、水土流失小流域治理。大力提升“生态美”超市，巩固农药集中配送成果，实施有机肥替代化肥行动，推进畜禽养殖废弃物处置和资源化利用，力争农村污水治理PPP项目（一期）建成运行。全面落实林长制，扎实推进林业增绿增效行动。深入开展松材线虫病治理专项行动，强化治理工作的责任落实，确保黄山松和森林资源安全。严格落实耕地保护主体责任，大力推进补充耕地建设。深化重点领域、重点行业节能减排工作，加强固体废弃物处置，抓好秸秆禁烧和综合利用、城市餐饮油烟综合整治、建设工地扬尘污染防治、烟花爆竹禁限放和高污染燃料禁燃工作，确保空气质量继续保持全国领先，让黄山大地天更蓝、山更绿、水更清、环境更优美。

（四）强力推进新型工业化。

坚持工业强市、产业兴市，深入实施新型工业化“5542”行动计划，大力推动制造业高质量发展，力争规上工业增加值增长8.5%。

培育壮大主导产业。常态化推进“四送一服”双千工程，严格落实市领导联系市级队企业制度，稳定企业预期、提振发展信心，力争市级队企业产值增速高于规上工业产值增速2个百分点以上。突出抓好4个省级新型工业化发展基地和15个省级产业集群专业镇建设，力争四大主导产业产值增速高于规上工业产值增速2个百分点以上。深入推进“三重一创”，做大做强省级文化旅游战新基地，加快推进徽州区先进结构材料省级重大新兴产业工程建设，抓好2个市级战新基地建设，力争全市战略性新兴产业产值增速高于规上工业产值增速2个百分点。

加快开发区改革和创新发展。支持市经济开发区争创国家高新技术产业开发区、歙县经济开发区争创国家级开发区，全市开发区经营收入、规上工业增加值、固定资产投资均增长10%。全面实施全市开发区主导产业发展规划，做大做强传统优势产业，加快建设现代特色产业。严守开发区生态保护红线，加大闲置土地清理和“僵尸企业”处置力度，持续开展腾笼换鸟，推动各开发区集聚发展、跨越发展。推进市场化运营改革，推广“管委会+公司”模式，激活各类开发区发展活力。市经济开发区要坚持“生产、生活、生态”融合发展，着力壮大绿色食品、智能制造、电子信息三大主导产业，突出抓好未来科技城、新兴产业园、徽艺小镇、绿色食品园建设，规划建设市奥体中心和会展中心，确保菲英二期、中鼎二期等项目建成运营，全年开工建设超10亿元项目8个，主要经济指标实现两位数增长。

大力推进科技创新。深入实施技术改造全覆盖、制造业与互联网融合发展、专精特新培育三大工程，培育一批科技创新、孵化平台，建立健全企业技改服务体系，全年实施技术改造项目150个以上，技改投资增长8%以上；新增国家贯标评定企业1家以上，省级智能工厂1家、数字化车间2家，市级专精特新企业30家。开展质量提升行动，推进“质量强市”建设。实施服务型制造三年行动计划，推动先进制造业和现代服务业深度融合。深入开展“双百双行”产学研对接活动，新组建院士工作站1家，引进高层次人才团队2个以上，新增高新技术企业6家，全年高新技术产业增加值增幅高于规上工业增加值增幅2个百分点以上。

（五）加快推进服务业转型升级。

以纪念邓小平同志“黄山谈话”40周年为新起点，深入实施“旅游+”战略，全力提升旅游国际化水平，加快构建以旅游业为主体、多业融合的现代服务业发展新格局。

深入实施旅游“品质革命”。切实抓好皖南国际文化旅游示范区绿色主导产业集群建设，大力推进旅游“品质革命”行动计划，加快全域旅游示范区创建，推动旅游业转型升级、提质增效，力争游客接待量增长14%，入境游客增长9%，旅游总收入增长15%，过夜登记游客超1000万人。实施黄山东部大开发战略，加快东海景区游步道、客运索道和东大门综合服务中心建设，拉开东黄山国际小镇建设框架，加大小镇国际化招商力度。推动齐云山、牯牛降、新安江山水画廊创建5A级景区，太平湖争创国家级旅游度假区。制定实施全域民宿业发展规划，支持民宿产业规范发展，打响徽州民宿品牌。大力发展研学旅游，系统开发摄影写生、文化体验、赏花晒秋等多元业态。加快发展运动休闲健康产业，打造登山、徒步、骑行、营地、马拉松、低空飞行、水上运动等特色产品。突出发展伴手礼产业。持续加大“梦幻黄山·礼仪徽州”城市品牌形象推广力度，创新办好黄山旅游节、国际登山大会、黟县山地车节等赛事活动，抓好高铁沿线和通航城市旅游营销，巩固提升香港、台湾、韩国及东南亚市场，积极开拓欧美市场。规划建设全域旅游营运中心，推进旅游标识设施建设和高速服务区旅游化改造，加快通景公路和旅游快线建设。推进旅游管理领域相对集中行政处罚权试点全覆盖。推广“一部手机游黄山”，开启黄山幸福旅游、美丽旅游、智慧旅游、信用旅游新体验。

大力发展现代服务业。创新管理体制机制，做大做强黄山现代服务业产业园。全面完成商山路、宾虹路改造提升，进一步拉开园区框架。建成市文化艺术中心、昌仁医养综合体、锋速车技馆，整合文博馆群和文创小镇等资源开展市场化运营，打造黄山“研学旅行第一站”，新签亿元以上项目7个，协议投资额超30亿元。加快20家省市服务业集聚区建设，扎实推进黟县省级服务业综合改革试点。深入实施新安医学振兴工程，加快发展大健康产业和中医药产业。大力培育规（限）上服务业企业，加快传统商圈、特色街区提档升级，积极发展电商、文博、会展等新型服务业。加强房地产市场监管，促进房地产市场平稳健康发展。全力抓好100个重点文化产业项目建设，力争新增省级文化产业示范基地5家、省级服务业集聚区（示范区）1家，服务业增加值增幅高于地区生产总值增幅。

（六）大力实施乡村振兴战略。

编制实施乡村振兴战略规划，着力打造“六有”新乡村，推动乡村振兴走在全省乃至全国前列。

深化农村综合改革。全面开展农村集体经济组织成员身份确认工作，加快经营性资产股份合作制改革，高质量推进集体产权制度改革工作。积极推动农村“三变”改革，力争全市一半以上的村完成改革任务。充分发挥农村产权交易市场体系功能，力争全年交易额突破2亿元。积极探索农村宅基地“三权”分置改革，整县推进歙县、休宁县盘活闲置农房试点。抓好“一事一议”财政奖补项目、国家扶持村集体经济发展试点和黄山区国家级农村综合性改革试点工作。

大力发展精致农业。深入实施农业产业化“826”行动计划，加快打造农产品加工产业集群，大力培育新型经营主体，力争规上农产品加工企业产值增长10%，全年新发展农业产业化联合体10个。深入推进茶业全产业链有机化试点示范，着力提升茶叶质量安全水平，茶叶一产产值达36亿元。继续开展重点区域油菜高产创建示范片建设，支持黟县大力发展“五黑”产业，因地制宜发展黄山贡菊、干鲜果、食用菌、覆盆子、皖南花猪、黄山中蜂等特色种养业，加大黄山毛峰、太平猴魁、祁门红茶、黄山泉水鱼、黄山臭鳜鱼等区域公共品牌创建与保护。加强农产品全程质量追溯体系建设。持续抓好非洲猪瘟防控工作。加快推进农村三次产业融合发展，深入实施休闲农业和乡村旅游精品工程，推进歙县蓝田·桃源田园综合体试点建设，继续办好中国休闲农业（黄山）峰会和黄山茶会。实施“互联网+”农产品出村工程，农产品电商销售额增长10%以上，让更多的黄山特色农产品畅响全国、畅销世界。

加快建设美丽乡村。学习借鉴浙江“千万工程”经验做法，高标准启动2019年度美丽乡村建设任务。强化乡镇（村）规划引领，实施乡镇（村）规划员培训工程。扎实开展农村人居环境整治三年行动，启动实施“五清一改”清洁家园行动，统筹推进农村“三大革命”，持续抓好“蓝色屋面”专项整治，巩固“大棚房”问题专项清理整治成果。完成世行贷款新农村建设项目。建成“四好农村路”扩面延伸工程327公里。健全自治、法治、德治相结合的乡村治理体系，以新时代文明实践中心（站）为平台，加强德育建设，提高农民素质，推动美丽乡村实现“外在美”与“内在美”的有机统一。

（七）切实提升城市建设管理水平。

以完善城市功能、提升城市品质为着力点，以“六城联创”和“城市双修”为抓手，大力推进智慧城市建设，深入开展“两治三改”三年行动，全力补短板、强内质、提水平，加快打造皖南现代化区域性中心城市。

突出抓好中心城区建设。创新推动城市建设高质量发展。统筹推进“一环三片”建设，着力抓好路网等基础设施建设，建成黎阳桥、八一大桥，开工建设阳湖桥，完成镇海桥维修加固和梅林大道改造提升，实施华山路、横江路等29条道路提升工程。全面提速屯溪老街滨江西路综合提升、花溪饭店地块改造和“徽州坊”文化旅游综合体建设，加快打造三江口城市核心商圈。持续推进省级优先发展公共交通示范城市创建，建设上塘公园、长干西路等4个公共停车场和智能管理系统。实施佩琅河、横关溪、霞塘河等新安江支流治理。推进城市排水防涝三年行动。加快市资源循环利用基地建设，启动城市污水厂污泥与餐厨垃圾处置PPP项目，建设市五水厂和南部城镇群水务调度中心，大幅提升中心城区综合承载力。

抓好区县城和特色小镇建设。加快市域南部屯、徽、休、歙同城步伐，推进城市基础设施、公共服务设施互联互通、共建共享。高水平规划建设区县城，有序推进农业转移人口市民化，力争户籍人口城镇化率提高1.3个百分点。强化产业支撑，增强辐射带动，巩固首批12个特色小镇建设成果，全面启动第二批市级特色小镇建设，突出抓好100个重点项目，力争全年完成投资超50亿元，新增省级以上特色小镇1-2个，特色小镇建设继续走在全省前列。

加快提升城市管理水平。抓好华为新型智慧城市云平台、科大讯飞智慧城市协同运营中心建设，全面提高智慧城市建设水平。强化城市规划管控，实施中心城区建筑立面风貌整治和重点路段亮化提升，推进美丽社区建设和交通秩序、市容环境等重点整治，全面提升城市管理科学化、精细化水平。广泛深入开展群众性精神文明创建活动，全面落实创建全国文明城市三年行动计划，深入实施市民素质、城市品质提升工程和“九九行动”，支持歙县争创全国县级文明城市、休宁县争创全省文明示范县，努力建设崇德向上、文化厚重、和谐宜居、人民满意的文明城市。

（八）加强保障和改善民生。

牢固树立以人民为中心的发展思想，多行惠民之举、多办利民之事，让人民群众有更多、更直接、更实在的获得感、幸福感、安全感。

扎实做好民生工作。认真组织实施民生工程，确保民生工程工作走在全省前列。实施就业优先政策，重点解决好高校毕业生、农民工、退役军人等群体就业，全年城镇新增就业2.1万人，城镇登记失业率控制在4.2%以内。深入实施“全民参保计划”，完善机关事业单位养老保险改革配套政策，建立城乡居民养老保险待遇确定和基础养老金正常调整机制，城镇企业职工养老、基本医疗、工伤、失业、生育五项保险参保人数达96.8万人次。加快城乡低保一体化，着力解决城市困难职工基本生活问题。大力发展社会福利、社会慈善事业。继续实施困难残疾人生活补贴、重度残疾人护理补贴制度，建立残疾儿童康复救助制度。支持社会力量兴办养老服务机构，力争全市“三级中心”覆盖率达60%。加强住房公积金建制扩面。严格落实棚改政策，加大征迁工作力度，改造城镇棚户区3946套，鼓励有条件的老旧小区加装电梯，让更多老百姓实现安居宜居。

大力发展社会事业。深入实施人才强市战略，加强新时代产业工人队伍建设，认真落实“人才10条”“技能人才15条”，推进“徽匠振兴工程”，全年引进高层次人才30人，新培养高技能人才1600人，新增省级博士后工作站1家。积极备战第45届世界技能大赛，力争再创佳绩。全面深化新时代教师队伍建设改革。继续实施学前教育行动计划，深入推进义务教育优质均衡试点。大力提升高中教育质量。推进职业教育资源整合和产教融合。办好特殊教育。支持黄山学院一流学科、硕士点建设。支持黄山电大建设开放大学。深入实施全民科学素质行动计划纲要，市科技馆建成开馆。持续开展历史建筑保护利用，支持徽州古城城墙申报世界文化遗产。深入推进徽州文化生态保护实验区建设，深化与故宫博物院合作，持续推进徽州文书申遗工程，办好第四届中国（黄山）非遗传统技艺大展。加快推进名镇名村名街志和特色志编纂工作。完善公共文化服务体系，推动县级媒体融合改革发展，实施广播电视制播覆盖提升工程。扎实推进健康黄山建设，推广基本公共卫生服务项目“两卡制”，规范医联体、医共体建设，提升县级医院综合服务能力，启动智慧医院建设。深化生育服务制度改革。积极创建全民运动健身模范市。启动第七次全国人口普查。充分发挥工会、共青团、妇联等人民团体的重要作用。健全农村留守儿童和妇女、老年人、残疾人关爱体系。做好民族、宗教、档案、地方志等工作。深入贯彻习近平强军思想，聚焦备战打仗，扎实做好军事斗争准备，进一步加强国防动员和后备力量建设。深化全民国防教育，推动军民融合深度发展，提升人民防空、双拥工作和退役军人服务管理水平。

加强和创新社会治理。坚持和发展新时代“枫桥经验”，深入推进各级综治中心标准化建设，不断提升社会治理社会化、法治化、专业化、智能化水平。深入推进“七五”普法，完善公共法律服务体系，做好人民调解、法律援助、社区矫正、安置帮教等工作。巩固拓展“深重促”专项行动成果，加大信访突出问题化解攻坚力度，完善矛盾纠纷多元化解机制。严格落实安全生产责任制，深入实施“铸安”行动和风险管控“六项机制”，严防各类重特大责任事故发生。加强应急管理，做好防汛抗旱、森林防火、地质灾害防治和气象、地震等工作，切实提升综合防灾减灾救灾能力。全面落实食品药品安全源头治理、分类监管、全程追溯等机制，争创国家食品安全示范城市。推进公共安全视频“雪亮工程”建设，完善立体化信息化社会治安防控体系，持续开展“守护平安”系列行动，纵深推进扫黑除恶专项斗争，努力建设更高水平的“平安黄山”。

**三、加强政府自身建设**

以政治建设为统领，以机构改革为动力，全面加强政府自身建设，加快转变政府职能，着力建设忠诚干净担当的干部队伍，打造为民务实高效清廉的服务型政府。

一要忠诚为政，坚定“两个维护”。始终把对党绝对忠诚作为最重要的政治纪律、最根本的政治规矩，深入学习贯彻习近平新时代中国特色社会主义思想，全面加强意识形态工作，扎实推进“两学一做”学习教育常态化制度化，深入开展“三查三问”，树牢“四个意识”，坚定“四个自信”，坚决维护习近平总书记党中央的核心、全党的核心地位，坚决维护党中央权威和集中统一领导。严格遵守党章党规党纪，认真贯彻《关于新形势下党内政治生活的若干准则》《中国共产党党内监督条例》，严格执行民主集中制，努力营造风清气正的良好政治生态。坚决贯彻落实省委省政府和市委各项重大部署，确保政令畅通、执行有力。

二要依法行政，坚持规范公正。认真履行法治政府建设职责，自觉在宪法和法律范围内履行职责、行使权力、开展工作。严格落实重大决策公众参与、专家论证、风险评估、合法性审查、集体讨论决定等程序，提升科学民主依法决策水平。加强政府立法工作，健全完善咨询、沟通、评估等机制，制定出台《黄山市餐厨垃圾管理办法》《黄山市林长制规定》。严格落实行政执法公示、执法全过程记录、重大执法决定法制审核“三项制度”，推进执法行为规范化建设，严格规范公正文明执法。落实重大决策出台前向人大及其常委会报告制度，加强与政协民主协商，广泛听取各民主党派、工商联和无党派人士意见，自觉接受人大及其常委会的法律监督、工作监督和政协的民主监督，严格落实人大及其常委会的决议决定和审议意见，提高人大代表议案、建议和政协提案办理质量。全面推进政务公开，让权力始终在阳光下运行。

三要高效施政，坚毅担当作为。大力倡导“马上就办、真抓实干”，深入推进机关效能建设，组织实施“数字政府”建设推进工程，优化行政资源配置，提高行政效率，构建统一协调、权威高效的行政服务机制。加强学习和调查研究，严格落实领导干部联系贫困村等制度，组织开展“大学习大调研大落实”活动，察实情、出实招、办实事、求实效。健全完善抓落实长效机制，严格规范督查检查考核，切实减少会议文件，让干部从繁杂的事务中解脱出来，把时间和精力用在谋发展、惠民生、抓落实上。全面落实《黄山市激励广大干部新时代新担当新作为实施细则（试行）》，旗帜鲜明地为敢于负责、勇于担当、善于作为、实绩突出的干部鼓劲撑腰，激励广大干部干字当头、干在实处，干出人民满意的新业绩。

四要廉洁从政，坚守纪律底线。严格落实《中国共产党廉洁自律准则》《中国共产党纪律处分条例》，切实履行全面从严治党主体责任和“一岗双责”，持续抓好政府系统党风廉政建设和反腐败斗争。严格执行中央八项规定精神及省委、市委实施细则，坚决反对形式主义、官僚主义，坚定不移纠“四风”、树新风，坚决整治群众身边的腐败和作风问题。认真抓好省委巡视、经济责任审计反馈问题整改，持续加强重点领域、关键环节权力运行制约监督，严格规范项目招投标、土地使用权与矿业权出让、国有产权交易、政府采购等行为，加强财政资金监管，推进经济责任审计监督全覆盖。坚持无禁区、全覆盖、零容忍，支持纪检监察机关依法依规履行职责，推动干部清正、政府清廉、政治清明。

各位代表！

百舸争流、奋楫者先，激荡时代、奋斗者胜。让我们紧密团结在以习近平同志为核心的党中央周围，在省委、省政府和市委的坚强领导下，以坚如磐石的信心、只争朝夕的劲头、坚忍不拔的毅力，锐意进取、扎实工作，为加快建设更美丽更富裕更文明的现代化新黄山不懈奋斗！
